# Association Between Revised Macronutrient Intake Ratios and Metabolic Syndrome

**DOI:** 10.3390/nu18162706

**Published:** 2026-08-19

**Authors:** Min Ju Kim, Jung Hyun Kwak

**Affiliations:** Department of Food Technology and Nutrition, Inje University, Gimhae 50834, Republic of Korea; sayme7725@naver.com

**Keywords:** metabolic syndrome, Korean Dietary Reference Intakes, acceptable macronutrient distribution range, macronutrient intake, KNHANES

## Abstract

**Background:** The 2025 Dietary Reference Intakes for Koreans (KDRIs) revised the acceptable macronutrient distribution ranges (AMDRs), but evidence regarding their association with metabolic syndrome (MS) is limited. **Methods:** We analyzed data from 14,768 Korean adults aged ≥19 years who participated in the Korea National Health and Nutrition Examination Survey (KNHANES) 2022–2024. Macronutrient intake ratios were classified according to the revised KDRIs. Multivariable logistic regression was used to estimate odds ratios (ORs) and 95% confidence intervals (CIs) after adjustment for demographic, socioeconomic, lifestyle, and dietary factors. Isocaloric substitution analyses estimated the associations of replacing 5% of energy from carbohydrate with fat or protein while maintaining total energy intake. **Results:** Overall adherence to all three AMDRs was not significantly associated with prevalent MS. Carbohydrate intake > 65%E and fat intake < 15%E were associated with higher odds of prevalent MS, whereas fat intake > 30%E and protein intake > 20%E were associated with lower odds. Significant sex interactions were observed for carbohydrate and fat intake categories, while no significant age interactions were found for the categorical analyses. Theoretical isocaloric substitution of 5%E from carbohydrate with fat or protein was associated with lower odds of prevalent MS, with significant interaction by sex for fat substitution and by age group for both fat and protein substitutions. **Conclusions:** In this cross-sectional analysis, higher carbohydrate and lower fat intake were associated with higher odds of prevalent MS, whereas higher fat and protein intake and theoretical isocaloric substitution of carbohydrate with fat or protein were associated with lower odds.

## 1. Introduction

A recent systematic review study estimated that approximately 1.54 billion adults worldwide were living with metabolic syndrome (MS) in 2023; the global prevalence of MS increased from 14.7% to 31.0% among women and from 9.0% to 25.7% among men between 2000 and 2023 [1]. According to the 2024 Korea Metabolic Syndrome Fact Sheet, the prevalence of MS increased from 27.74% before the COVID-19 pandemic to 29.69% after the pandemic [2]. MS is a major public health issue characterized by the coexistence of metabolic abnormalities such as central obesity, elevated blood pressure, impaired glucose regulation, hypertriglyceridemia, and reduced HDL-cholesterol levels [3]. These combined metabolic disturbances substantially increase the risk of developing Cardiovascular Disease and Type 2 Diabetes [4].

Several previous studies have reported associations between macronutrient intake and MS. Ha et al. reported that a low-carbohydrate diet was not associated with an increased risk of MS in Korean adults [5]. In addition, Lee et al. reported that higher protein intake was associated with lower risks of obesity, abdominal obesity, and MS, together with more favorable triglyceride and HDL-cholesterol profiles [6]. Consistent with these findings, another study by Lee et al. showed that a very-high-carbohydrate dietary pattern among Koreans was characterized by lower intakes of protein-rich foods, including meat, fish, eggs, beans, and dairy products, and was associated with several metabolic risk factors. The authors therefore highlighted the need to establish an optimal macronutrient distribution of carbohydrates, proteins, and fats for the prevention and management of metabolic diseases [7].

In the 2025 Dietary Reference Intakes for Koreans (KDRIs), the lower limit of the acceptable macronutrient distribution range (AMDR) for carbohydrate intake was revised from 55 to 65% to 50–65% of total energy intake, while the lower limit for protein intake was increased from 7 to 20% to 10–20% [8]. These revisions reflect emerging evidence suggesting that excessive carbohydrate-centered diets may be associated with increased risks of MS, cardiovascular disease, and mortality, as well as growing recognition of the importance of adequate protein intake for the prevention of sarcopenia and maintenance of metabolic health [8]. However, previous studies have mainly focused on the associations between specific nutrients or dietary patterns and MS, and evidence regarding the relationship between adherence to the newly revised macronutrient intake recommendations and MS risk remains limited [5,6,7]. Furthermore, sex- and age-specific differences in the relationship between macronutrient distribution and MS have not been sufficiently investigated. Therefore, the present study aimed to investigate the association between revised macronutrient intake ratios and MS among Korean adults using data from the Korea National Health and Nutrition Examination Survey (KNHANES) 2022–2024. In addition, subgroup analyses according to sex and age group were conducted.

## 2. Materials and Methods

### 2.1. Study Population

The data used in this study were obtained from the KNHANES 2022–2024, a nationally representative cross-sectional survey conducted by the Korea Disease Control and Prevention Agency. KNHANES consists of health interviews, health examinations, and nutrition surveys designed to assess the health and nutritional status of the Korean population. Among the 20,191 participants initially enrolled in KNHANES 2022–2024, individuals aged younger than 19 years were excluded (*n* = 2832). Participants with missing information on household income (*n* = 58), education level (*n* = 427), smoking status (*n* = 9), body mass index (BMI) (*n* = 296), total energy intake (*n* = 10), and physical activity variables including walking and muscle-strengthening exercise (*n* = 839) were additionally excluded.

**Figure 1:**
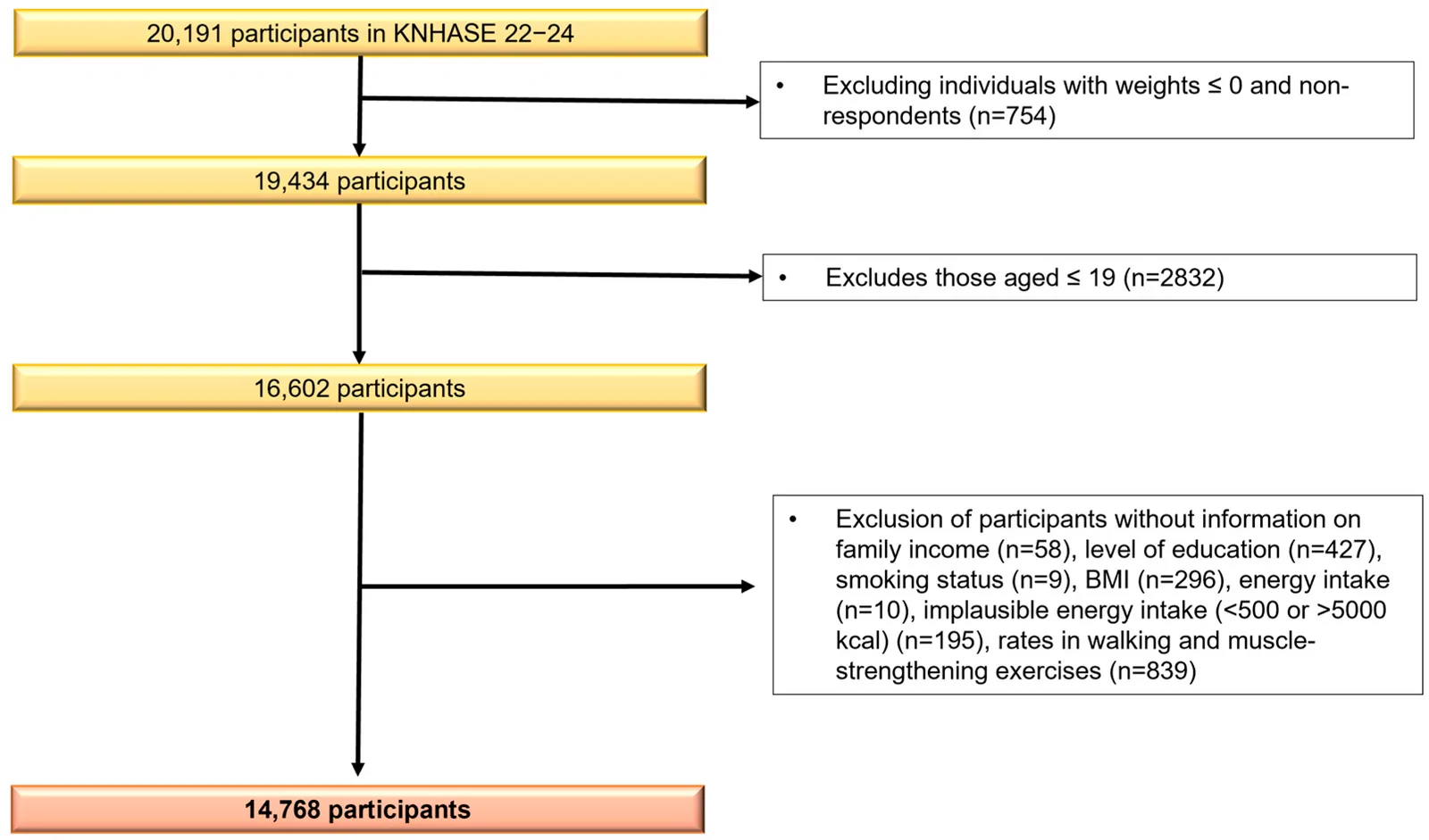
Flow chart of study population selection.

To minimize the influence of potentially implausible or extreme dietary intake reports, participants with total energy intakes of <500 or >5000 kcal/day (*n* = 195) were excluded. Participants with sampling weights ≤ 0 or non-respondents (*n* = 754) were also excluded. Consequently, a total of 14,768 adults aged 19 years or older were included in the final analysis. A flow chart of participant selection is presented in Figure 1.

### 2.2. Dietary Assessment and Macronutrient Intake Ratio

Because food frequency questionnaire (FFQ) data were not available in the present study, dietary intake was assessed using a single 24 h dietary recall method from the nutrition survey component of the KNHANES [9]. Trained interviewers collected detailed information on all foods and beverages consumed by participants during the day prior to the interview date (i.e., intake from two days before the survey) according to standardized protocols. Information on meal type, food ingredients, and portion sizes was obtained. Therefore, the macronutrient intake assessed in this study represents one-day reported dietary intake rather than habitual dietary intake. Nutrient intake was calculated using the Korean Food Composition Database developed by the Rural Development Administration, and daily energy and nutrient intakes were estimated based on standardized KNHANES procedures [10]. The intake ratios of carbohydrate, fat, and protein were calculated as the percentage of total energy intake contributed by each macronutrient. Macronutrient intake ratios were classified based on the acceptable macronutrient distribution ranges (AMDRs) of the Dietary Reference Intakes for Koreans (KDRIs): carbohydrate 50–65% of total energy intake, fat 15–30%, and protein 10–20%. Participants whose intake ratios met all three recommended ranges were classified into the “adequate ratio” group, whereas those outside any of the recommended ranges were classified into the “inadequate ratio” group. Additionally, carbohydrate intake ratios were categorized into <50%, 50–65%, and >65%; fat intake ratios into <15%, 15–30%, and >30%; and protein intake ratios into <10%, 10–20%, and >20%.

### 2.3. Definition of Metabolic Syndrome

MS was defined according to the revised National Cholesterol Education Program Adult Treatment Panel III (NCEP ATP III) criteria using Korean-specific waist circumference cutoffs [11]. Participants meeting three or more of the following criteria were classified as having MS: (1) abdominal obesity (waist circumference ≥ 90 cm for men and ≥85 cm for women), (2) elevated triglycerides (≥150 mg/dL or current treatment), (3) reduced HDL-cholesterol (<40 mg/dL for men and <50 mg/dL for women or current treatment), (4) elevated blood pressure (systolic blood pressure ≥ 130 mmHg, diastolic blood pressure ≥ 85 mmHg, or antihypertensive medication use), and (5) elevated fasting glucose (≥100 mg/dL or antidiabetic medication use).

### 2.4. Covariates

Potential confounding variables were selected based on previous literature and included demographic, socioeconomic, and lifestyle-related factors [5,6,7]. Age was treated as a continuous variable, and sex was categorized as male (reference) or female. BMI was calculated as weight in kilograms divided by height in meters squared (kg/m^2^) and categorized according to the Asia-Pacific criteria as underweight/normal (<23.0 kg/m^2^) (reference), overweight (23.0–24.9 kg/m^2^), and obese (≥25.0 kg/m^2^) [12]. Education level was classified into three categories: less than middle school graduate (reference), high school graduate, and college degree or higher. Household income was categorized into quartiles: low (reference), middle-low, middle-high, and high. Smoking status was categorized as never (reference), former, or current smoker. Alcohol consumption was classified into five categories according to drinking frequency during the past year: never (reference), past, current (≤1 time/month), current (2–4 times/month), and current (≥2 times/week). Physical activity variables included walking rate and strength-training rate. Walking rate was categorized as yes (≥5 days/week and ≥30 min/day) or no (reference), while strength-training rate was categorized as yes (≥2 times/week) or no (reference).

### 2.5. Statistical Analysis

All statistical analyses were conducted using complex survey procedures reflecting the stratified multistage probability sampling design and sample weights of KNHANES to provide nationally representative estimates. Continuous variables are presented as means ± standard errors (SE), and categorical variables are presented as weighted percentages (%). Differences in participant characteristics according to macronutrient intake ratio groups were assessed using survey *t*-tests for continuous variables and Rao–Scott chi-square tests for categorical variables. Multivariable logistic regression analyses were performed to evaluate the associations between macronutrient intake ratios and MS. Odds ratios (ORs) and 95% confidence intervals (CIs) were calculated. Model 1 was adjusted for age, sex, education level, household income, BMI, smoking status, alcohol consumption, total energy intake, walking rate, and strength-training rate. Model 2 was further adjusted for individual macronutrient intake and dietary fiber intake. Macronutrient intake was categorized according to the AMDR based on the percentage of total energy derived from each macronutrient. In the multivariable models, macronutrients included as covariates were expressed as absolute daily intake (g/day). When carbohydrate AMDR category was the exposure, protein and fat intakes (g/day) were adjusted for; when fat AMDR category was the exposure, carbohydrate and protein intakes (g/day) were adjusted for; and when protein AMDR category was the exposure, carbohydrate and fat intakes (g/day) were adjusted for. The macronutrient corresponding to the exposure was not additionally included as a covariate in the same model. Dietary fiber intake was included as absolute daily intake (g/day). Isocaloric substitution analyses were performed to estimate the theoretical cross-sectional association corresponding to replacing carbohydrate with fat or protein while maintaining total energy intake. Carbohydrate percentage was omitted from the model, whereas percentages of energy from fat and protein and total energy intake were simultaneously included. The logistic regression model was specified as follows:

$$logit\{P(MS\;=\;1)\}\;=\;\beta_{0}\;+\;\beta_{1}(\frac{Fat\%E}{5})\;+\;\beta_{2}(\frac{Protein\%E}{5})\;+\;\beta_{3}(Total\;energy\;intake)\;+\;\sum_{k=1}^{p}\gamma_{k}Z_{k}$$

The isocaloric substitution analyses were also performed using the KNHANES complex survey design, incorporating strata, PSUs, and sampling weights.

Restricted cubic spline analyses with four knots placed at the 5th, 35th, 65th, and 95th percentiles were additionally performed to examine potential nonlinear associations of fat and protein energy percentages with prevalent MS. Nonlinearity was assessed by jointly testing the spline terms beyond the linear component. Analyses were stratified by sex and age group, with adjustment for potential confounding variables. 

BMI-adjusted models were considered the primary models, while sensitivity analyses were additionally conducted without BMI adjustment to evaluate the potential influence of overadjustment related to adiposity. 

Multicollinearity among the exposure and covariates included in each exposure-specific multivariable model was assessed using variance inflation factors (VIFs), with a VIF ≥ 10 considered indicative of substantial multicollinearity.

Potential effect modification by sex and age group (19–39, 40–64, and ≥65 years) was formally evaluated by including interaction terms between sex or age group and each macronutrient intake category in the survey-weighted multivariable logistic regression models. Interactions of sex and age group with the continuous fat and protein terms were also tested in the isocaloric substitution models. The statistical significance of interactions involving categorical variables was evaluated using joint Wald tests, and the corresponding *p* values for interaction were reported. Subgroup-specific estimates were interpreted as evidence of potential effect modification only when supported by statistically significant interaction tests.

Sex- and age-stratified analyses were considered exploratory. To account for multiple comparisons, the Benjamini–Hochberg false discovery rate (FDR) procedure was additionally applied as a sensitivity analysis, separately for the categorical macronutrient analyses and the isocaloric substitution analyses.

All statistical analyses were conducted using SAS software (version 9.4; SAS Institute, Cary, NC, USA), and a two-sided *p*-value < 0.05 was considered statistically significant.

## 3. Results

### 3.1. General Characteristics of Participants According to Revised Macronutrient Intake Ratio

A total of 14,768 Korean adults were included in the analysis, of whom 4176 participants were classified into the adequate macronutrient ratio group and 10,592 into the inadequate ratio group. Participants in the inadequate ratio group had significantly higher total energy intake and fat intake, but lower carbohydrate intake compared with those in the adequate ratio group (all *p* < 0.001) (Table 1). Regarding socioeconomic factors, the inadequate ratio group had a higher proportion of participants with lower educational attainment and lower household income (both *p* < 0.01). In terms of lifestyle factors, frequent alcohol consumption (≥2 times/week) was more prevalent in the inadequate ratio group (*p* < 0.001). However, no significant differences were observed in BMI distribution, smoking status, physical activity, or prevalence of MS between the two groups. Among men, HDL-C levels were higher in the AMDR-inadequate group than in the adequate group (52.1 ± 0.2 vs. 50.9 ± 0.3 mg/dL, *p* = 0.002), whereas no difference was observed among women (63.2 ± 0.3 vs. 63.1 ± 0.4 mg/dL, *p* = 0.686). The prevalence of elevated triglycerides did not differ between the groups (24.7 ± 0.5% vs. 26.0 ± 0.8%, *p* = 0.167), whereas elevated fasting glucose was more prevalent in the AMDR-inadequate group (35.3 ± 0.6% vs. 33.4 ± 0.8%, *p* = 0.043).

### 3.2. Associations Between General Characteristics and Metabolic Syndrome

Females had a significantly lower odds of MS compared with males (OR: 0.59, 95% CI: 0.51–0.67) (Table 2). Increasing age was positively associated with the odds of MS (OR: 1.05, 95% CI: 1.04–1.05). Obesity showed the strongest association with MS; compared with participants with BMI < 23 kg/m^2^, overweight individuals had 3.87-fold higher odds of MS, while obese individuals had 19.07-fold higher odds.

Current smokers exhibited significantly increased odds of MS (OR: 1.62, 95% CI: 1.37–1.92). In contrast, regular walking activity and strength training were inversely associated with the odds of MS. Participants who engaged in walking ≥ 5 days/week had 17% lower odds of MS (OR: 0.83, 95% CI: 0.76–0.92), whereas those performing strength training ≥ 2 times/week had 42% lower odds (OR: 0.58, 95% CI: 0.51–0.65).

### 3.3. Association Between Revised Macronutrient Distribution and Metabolic Syndrome

No significant association was observed between overall adequate/inadequate macronutrient ratio classification and MS after multivariable adjustment (Table 3). However, specific macronutrient intake ratios were significantly associated with the odds of prevalent MS.

Participants consuming more than 65% of total energy from carbohydrates had significantly higher odds of MS compared with those consuming 50–65% carbohydrates (Model 2 OR: 1.14, 95% CI: 1.01–1.30). In contrast, a fat intake ratio greater than 30% was associated with lower odds of MS (OR: 0.83, 95% CI: 0.71–0.92), whereas fat intake below 15% was associated with higher odds of prevalent MS (OR: 1.25, 95% CI: 1.10–1.43). For protein intake, participants consuming more than 20% of total energy from protein showed significantly lower odds of MS (OR: 0.78, 95% CI: 0.67–0.92), while protein intake below 10% was not significantly associated with MS.

In sensitivity analyses excluding BMI from the multivariable models, the overall pattern of associations was generally consistent with that of the BMI-adjusted models (Table S2). Overall AMDR inadequacy remained unassociated with prevalent MS (BMI-adjusted: OR, 1.01; 95% CI, 0.91–1.13; BMI-excluded: OR, 1.01; 95% CI, 0.92–1.11). The higher odds associated with carbohydrate intake > 65%E and fat intake < 15%E, as well as the lower odds associated with fat intake > 30%E, persisted after BMI was excluded. In contrast, the inverse association for protein intake > 20%E was attenuated from 0.78 (95% CI, 0.67–0.92) in the BMI-adjusted model to 0.87 (95% CI, 0.76–1.01) in the BMI-excluded model and was no longer statistically significant.

### 3.4. Sex-Specific Associations Between Macronutrient Distribution and Metabolic Syndrome

Sex-stratified analyses revealed stronger associations among females. In women, fat intake below 15% was associated with increased odds of MS (OR: 1.40, 95% CI: 1.16–1.68), whereas fat intake above 30% was associated with reduced odds (OR: 0.73, 95% CI: 0.59–0.92) (Table 4). Additionally, protein intake above 20% was associated with lower odds of prevalent MS in women (OR: 0.73, 95% CI: 0.55–0.96).

Among men, only higher protein intake (>20%) was associated with lower odds of prevalent MS (OR: 0.80, 95% CI: 0.64–0.99). No statistically significant association was observed between carbohydrate intake ratio and MS in either sex, although women consuming more than 65% carbohydrate tended to have higher odds of prevalent MS.

Formal interaction analyses showed significant interactions between sex and carbohydrate intake category (*p* for interaction < 0.001) and between sex and fat intake category (*p* for interaction = 0.029), whereas the interaction between sex and protein intake category was not significant (*p* for interaction = 0.732) (Table S4). In the FDR sensitivity analysis of the sex-stratified categorical comparisons, two associations among women remained statistically significant after correction for multiple testing: carbohydrate intake > 65% of total energy (FDR-adjusted *p* = 0.037) and fat intake < 15% of total energy (FDR-adjusted *p* = 0.009) (Table S5).

### 3.5. Age-Specific Associations Between Macronutrient Distribution and Metabolic Syndrome

Age-stratified analyses demonstrated that the associations between macronutrient distribution and MS varied across age groups (Table 5). High carbohydrate intake (>65%) was also associated with higher odds of prevalent MS in adults aged 19-39 years (OR: 1.63, 95% CI: 1.08–2.43). Low fat intake (<15%) was consistently associated with higher odds of prevalent MS across all age groups, with the strongest association observed in younger adults (OR: 1.62, 95% CI: 1.02–2.59). Conversely, higher fat intake (>30%) was associated with lower odds of prevalent MS, particularly among middle-aged adults aged 40–64 years (OR: 0.69, 95% CI: 0.59–0.85). Higher protein intake (>20%) was associated with lower odds of MS among older adults aged ≥65 years (OR: 0.67, 95% CI: 0.49–0.92).

In contrast, no significant interactions were observed between age group and carbohydrate (*p* for interaction = 0.832), fat (*p* for interaction = 0.089), or protein intake category (*p* for interaction = 0.631) (Table S4). In the age-stratified analyses, fat intake > 30% of total energy among adults aged 40–64 years remained statistically significant after FDR correction (FDR-adjusted *p* = 0.009) (Table S5).

### 3.6. Isocaloric Substitution Analysis Stratified by Sex and Age Group

A theoretical isocaloric substitution of 5% of energy from carbohydrate with fat or protein was associated with lower odds of MS in the overall population (Table 6). The inverse association for the theoretical substitution of carbohydrate with fat was observed in both men and women and was particularly evident among adults aged 40–64 years. In contrast, the theoretical substitution of carbohydrate with protein was significantly associated with lower odds of MS in men and among participants aged 19–39 years and ≥65 years, whereas no significant associations were observed in women or among adults aged 40–64 years. Restricted cubic spline analyses showed no significant evidence of nonlinearity for either fat (*p* for nonlinearity = 0.582) or protein (*p* for nonlinearity = 0.190) energy percentage, supporting the use of linear continuous terms in the isocaloric substitution models.

In the isocaloric substitution analyses, a significant interaction was observed between sex and the theoretical substitution of carbohydrate with fat (*p* for interaction = 0.022), whereas the interaction between sex and the substitution of carbohydrate with protein was not significant (*p* for interaction = 0.190). Significant interactions with age group were observed for the theoretical substitution of carbohydrate with both fat (*p* for interaction = 0.007) and protein (*p* for interaction = 0.025), indicating heterogeneity in these statistical substitution associations across age groups (Table S4).

In addition, four of the 10 subgroup-specific associations remained statistically significant after FDR correction: carbohydrate-to-protein substitution among men (FDR-adjusted *p* = 0.011), carbohydrate-to-fat substitution among women (FDR-adjusted *p* = 0.011), carbohydrate-to-protein substitution among adults aged 19–39 years (FDR-adjusted *p* = 0.013), and carbohydrate-to-fat substitution among adults aged 40–64 years (FDR-adjusted *p* ≤ 0.001) (Table S5).

### 3.7. Associations of Mutually Exclusive Macronutrient Intake Patterns with Metabolic Syndrome

The mutually exclusive macronutrient patterns were overall significantly associated with prevalent MS (global Wald χ^2^ = 27.13, df = 7, *p* = 0.0003) (Table S9). Compared with participants whose carbohydrate, fat, and protein intakes were all within the AMDRs, those with a high-carbohydrate–low-fat pattern had higher odds of prevalent MS (OR, 1.30; 95% CI, 1.12–1.50), whereas those with a low-carbohydrate–high-fat pattern had lower odds (OR, 0.81; 95% CI, 0.67–0.98). The other mutually exclusive patterns were not significantly associated with prevalent MS.

## 4. Discussion

In this nationally representative study using KNHANES 2022–2024 data, we investigated the association between revised macronutrient intake ratios and MS among Korean adults. The major findings were as follows: (1) overall adherence to the revised AMDR was not associated with MS prevalence; (2) excessive carbohydrate intake (>65% of total energy) and very low fat intake (<15% of total energy) were associated with higher odds of MS; (3) higher fat intake (>30%) and higher protein intake (>20%) were associated with lower odds of MS; (4) isocaloric substitution analyses suggested that a theoretical 5%E replacement of carbohydrate with fat or protein was associated with lower odds of prevalent MS; and (5) formal interaction analyses indicated heterogeneity by sex or age group for some, but not all, associations.

The present findings suggest that the balance of individual macronutrient intake ratios may be more important than simply categorizing diets as adequate or inadequate according to overall recommended ranges. Although the overall macronutrient adequacy classification was not significantly associated with prevalent MS, analyses of mutually exclusive macronutrient patterns demonstrated heterogeneity within the original inadequate category. Compared with participants meeting all three AMDRs, those with a high-carbohydrate–low-fat pattern had higher odds of prevalent MS, whereas those with a low-carbohydrate–high-fat pattern had lower odds. These opposing associations suggest that combining distinct macronutrient deviation patterns into a single inadequate category may obscure pattern-specific associations with prevalent MS. This finding is consistent with previous studies conducted in Asian populations, where excessive carbohydrate intake has been associated with insulin resistance, elevated triglyceride concentrations, and abdominal obesity [13,14]. Korean dietary patterns are traditionally characterized by high consumption of refined carbohydrate-based foods such as white rice, which may contribute to unfavorable metabolic outcomes when consumed excessively [14]. High carbohydrate intake may increase postprandial glucose and insulin responses, promote hepatic de novo lipogenesis, and elevate circulating triglyceride levels, thereby increasing the risk of metabolic abnormalities [15].

Interestingly, low fat intake (<15% of total energy) was consistently associated with higher odds of MS across nearly all subgroups. Traditionally, low-fat diets have been recommended for cardiovascular and metabolic health; however, recent evidence suggests that excessively restricting fat intake may adversely affect metabolic regulation [16]. Adequate dietary fat intake contributes to satiety, maintenance of cell membrane function, hormone synthesis, and absorption of fat-soluble vitamins [8]. Furthermore, replacing dietary fat with refined carbohydrates may worsen insulin resistance and dyslipidemia [17]. In the present study, participants with fat intake above 30% showed lower odds of MS, particularly among women and middle-aged adults, suggesting an inverse cross-sectional association between moderate-to-higher fat intake and prevalent MS. An important consideration is that the observed inverse association between higher fat intake and MS should not be interpreted as supporting greater fat intake as a dietary recommendation. The metabolic effects of dietary fat are likely to depend not only on the amount consumed but also on the food sources and fatty acid composition. According to the 2022 KNHANES Statistics, the major food sources of dietary fat differ considerably by age and sex [18]. Younger adults obtain a greater proportion of dietary fat from processed and convenience foods such as fried chicken, ramen, hamburgers, and snacks, whereas older adults consume relatively more fat from foods such as milk, eggs, soybean products, and mixed grains. These findings suggest that the health effects of dietary fat may vary according to dietary patterns and food sources rather than total fat intake alone. Therefore, future studies should investigate not only total fat intake but also the quality of dietary fat and its food sources when evaluating the association between dietary fat and MS.

Higher protein intake (>20% of total energy) was also inversely associated with MS, especially among women and older adults. Protein-rich diets may improve satiety and help preserve skeletal muscle mass, which is particularly important in aging populations [19]. Increased protein intake may additionally improve glucose homeostasis through enhanced insulin sensitivity and reduced postprandial glycemic responses [20]. The stronger inverse association observed among older adults may reflect the importance of adequate protein intake for maintaining muscle mass and metabolic health during aging.

Sensitivity analyses excluding BMI yielded findings generally consistent with the primary analyses for carbohydrate and fat intake, suggesting that these associations were not materially dependent on BMI adjustment. However, the inverse association between protein intake > 20%E and prevalent MS was attenuated and became statistically non-significant after BMI was excluded. This finding suggests that the estimated association for high protein intake may be sensitive to the inclusion of BMI in the model. Given the cross-sectional design, the temporal relationships among macronutrient composition, BMI, and MS cannot be established, and BMI may function as a confounder, mediator, or correlate of dietary composition. Therefore, the inverse association observed for high protein intake should be interpreted cautiously.

The subgroup analyses demonstrated that the associations between macronutrient distribution and MS differed by sex and age. Women appeared to be more sensitive to dietary fat and protein composition than men. Hormonal differences, body fat distribution, and sex-specific metabolic responses may partly explain these findings [21]. Although carbohydrate intake > 65%E was associated with higher odds of prevalent MS among adults aged 19–39 years, the absence of a significant age interaction suggests that this finding should not be interpreted as evidence of an age-specific association. Younger adults may be particularly vulnerable to Westernized dietary patterns characterized by high intake of refined carbohydrates and sugar-sweetened foods combined with reduced physical activity [22]. These findings suggest potential sex- and age-specific differences in the cross-sectional associations between macronutrient distribution and prevalent MS, which warrant further prospective investigation. Several mechanisms may explain the observed associations between macronutrient distribution and MS. Excessive carbohydrate intake can induce repeated hyperglycemia and hyperinsulinemia, promoting visceral fat accumulation and dyslipidemia [16]. Conversely, adequate intake of dietary fat and protein may improve satiety regulation, reduce glycemic variability, and support healthy body composition [17,23]. Moreover, dietary fat quality may also play an important role; unsaturated fatty acids have been associated with improved lipid metabolism and insulin sensitivity [24]. Therefore, future studies should further investigate not only macronutrient quantity but also macronutrient quality and food sources.

Our isocaloric substitution analysis provides additional insight beyond the primary association between macronutrient intake and MS by providing theoretical isocaloric substitution estimates for carbohydrate with other macronutrients while holding total energy intake constant. In the overall population, replacing 5% of energy from carbohydrate with either fat or protein was associated with lower odds of MS, suggesting that the observed cross-sectional association with carbohydrate intake may differ according to the macronutrient theoretically substituted for carbohydrate. The inverse association observed when carbohydrate was replaced with fat is generally consistent with previous intervention studies demonstrating that replacing carbohydrates with unsaturated fat under isocaloric conditions improves triglyceride concentrations, HDL cholesterol, insulin sensitivity, and glycemic control, thereby reducing cardiometabolic risk [25,26].

Similarly, replacing carbohydrates with protein was associated with lower odds of MS in the overall population, particularly among men and participants aged 19–39 years and ≥65 years. Previous randomized controlled trials and meta-analyses have shown that isocaloric higher-protein diets improve body composition, preserve lean mass, and reduce triglyceride concentrations compared with higher-carbohydrate diets, with some studies also reporting improvements in insulin-related metabolic outcomes [27,28,29,30].

Interestingly, the associations differed according to sex and age. Replacing carbohydrate with fat showed relatively consistent inverse associations across both sexes and was most evident among middle-aged adults, whereas carbohydrate replacement with protein appeared to be primarily among men, younger adults, and older adults. These differences may reflect age- and sex-related variations in insulin sensitivity, skeletal muscle metabolism, hormonal regulation, and dietary protein requirements [21,30]. Further prospective studies are warranted to validate these subgroup-specific associations.

Importantly, the isocaloric substitution model represents a theoretical statistical comparison rather than an observed dietary change. Therefore, the lower odds of prevalent MS associated with the theoretical replacement of carbohydrate with fat or protein should not be interpreted as evidence that individuals who actively changed their macronutrient intake would experience a reduction in MS. Furthermore, our findings should not be interpreted as recommending the replacement of carbohydrate with any type of fat or protein, as the associations related to isocaloric macronutrient substitution may also depend on the quality of the macronutrient sources involved. Previous studies have consistently reported that replacing carbohydrates with unsaturated fats or plant-based protein sources is associated with more favorable cardiometabolic outcomes than replacement with saturated fats or processed animal proteins [31,32]. Prospective studies and dietary intervention trials are needed to determine whether actual changes in macronutrient composition influence the development of MS.

Finally, the application of an isocaloric substitution model represents an important strength of the present study because it provides theoretical estimates of the cross-sectional association corresponding to substitution of one macronutrient for another while holding total energy intake constant. This approach provides complementary information beyond analyses evaluating individual macronutrients independently.

Formal interaction analyses provided evidence that sex modified the associations of carbohydrate and fat intake categories with prevalent MS, whereas no evidence of interaction by sex was observed for protein intake category. Although some associations reached statistical significance only within specific age strata, these findings should not be interpreted as evidence that the categorical associations differed by age. In the isocaloric substitution analyses, however, significant interactions with age group were observed for the theoretical substitution of carbohydrate with both fat and protein, suggesting that the statistical substitution associations may vary across age groups These findings should be interpreted cautiously given the cross-sectional design and the exploratory nature of the subgroup analyses.

Consistent with this consideration, FDR sensitivity analyses showed that only three of 30 categorical subgroup comparisons and four of 10 substitution subgroup comparisons remained statistically significant after correction for multiple testing. These findings indicate that some nominally significant subgroup associations may reflect multiple testing and should therefore be regarded as exploratory. Prospective studies are needed to confirm whether these potential subgroup differences are reproducible.

This study has several strengths. First, we used nationally representative KNHANES data, allowing the findings to be generalized to the Korean adult population. Second, we simultaneously evaluated carbohydrate, fat, and protein intake ratios while adjusting for multiple socioeconomic and lifestyle-related confounders. Third, subgroup analyses according to sex and age provided additional insights into population-specific associations.

However, several limitations should also be considered. First, because this study had a cross-sectional design, causal relationships between macronutrient intake ratios and MS cannot be established. In addition, reverse causation cannot be excluded, as participants with existing metabolic abnormalities or previous medical advice may have modified their dietary intake before the survey. Second, dietary intake was assessed using a single self-reported 24 h recall, which may be subject to recall bias and day-to-day within-person variation. Because repeated dietary measurements were not available, individual-level usual macronutrient intake could not be estimated. Consequently, measurement error and nondifferential misclassification across the AMDR categories may have occurred, potentially attenuating the observed associations toward the null (regression dilution). Therefore, the AMDR classifications should be interpreted as reflecting one-day reported intake rather than habitual dietary patterns. Third, this study focused on the relative energy contributions of carbohydrates, fats, and proteins according to the AMDRs and did not account for differences in macronutrient quality or food sources. For example, refined carbohydrates and whole grains may have different metabolic implications, while saturated, monounsaturated, and polyunsaturated fatty acids may have distinct associations with metabolic health. Protein sources, such as plant- and animal-based proteins, may also differ in their metabolic implications. Therefore, the observed associations should be interpreted as reflecting differences in macronutrient energy distribution rather than the quality or specific food sources of these macronutrients. Future studies should jointly consider macronutrient quantity and quality, including carbohydrate sources and quality, fatty acid composition, and protein sources, to better characterize their associations with metabolic health. Fourth, although the isocaloric substitution models accounted for the interdependence of macronutrient energy contributions by estimating theoretical substitutions among macronutrients, they do not constitute a formal compositional data analysis based on log-ratio transformations. Therefore, residual limitations related to the compositional nature of macronutrient intake cannot be excluded. Future studies using formal compositional data-analysis approaches may further clarify associations between the relative macronutrient composition of the diet and metabolic health. Fifth, because BMI is strongly correlated with waist circumference, a component of the MS definition, adjustment for BMI may have attenuated part of the association between macronutrient intake and MS. However, sensitivity analyses excluding BMI yielded broadly similar estimates, suggesting that the main findings were robust to BMI adjustment. Sixth, residual confounding by unmeasured lifestyle or genetic factors may still exist despite extensive statistical adjustment. Finally, although formal interaction tests were conducted and FDR correction was applied as a sensitivity analysis, the large number of subgroup comparisons increases the possibility of chance findings. Therefore, subgroup-specific results, particularly those that did not remain significant after FDR correction, should be considered exploratory and require confirmation in future studies.

## 5. Conclusions

In conclusion, in this cross-sectional analysis based on one-day reported dietary intake, higher carbohydrate intake and very low fat intake were associated with higher odds of prevalent MS among Korean adults, whereas higher fat and protein intake ratios were associated with lower odds. In the context of the revised 2025 KDRIs, the current AMDRs of 50–65%E for carbohydrate, 10–20%E for protein, and 15–30%E for fat provide practical reference ranges for interpreting macronutrient distribution. Our findings suggest that attention to specific macronutrient distributions, rather than overall AMDR compliance alone, may be informative when evaluating dietary patterns associated with metabolic health. Formal interaction analyses indicated heterogeneity by sex or age group for some, but not all, associations; therefore, these exploratory findings should not be interpreted as establishing subgroup-specific optimal intake ranges. These findings also should not be interpreted as evidence of causal effects or habitual adherence to the KDRI AMDRs. Future prospective and intervention studies using repeated dietary assessments are needed to confirm these associations and to determine whether sex- and age-specific macronutrient recommendations beyond the current KDRIs are warranted.

## Figures and Tables

**Table 1:** Participants’ characteristics by revised macronutrient intake ratio among Korean adults in the KNHANES (2022–2024).

Characteristics	Adequate Ratio Group (*n* = 4176)	Inadequate Ratio Group (*n* = 10,592)	*p*-Value
Sex (% (SE))			0.007
Male	52.0 (0.8)	49.3 (0.5)	
Female	48.0 (0.8)	50.7 (0.5)	
Age (years, mean ± SE)	48.6 ± 0.4	49.1 ± 0.3	0.139
Total energy intake (kcal/day, mean ± SE)	1805.0 ± 12.1	1865.0 ± 9.8	<0.001
Carbohydrate intake (g/day, mean ± SE)	265.7 ± 1.8	253.7 ± 1.3	<0.001
Protein intake (g/day, mean ± SE)	70.3 ± 0.5	71.6 ± 0.5	0.051
Fat intake (g/day, mean ± SE)	48.6 ± 0.4	52.0 ± 0.5	<0.001
BMI (kg/m^2^, % (SE))			0.848
Underweight and normal (<23.0)	41.1 (0.9)	40.5 (0.5)	
Overweight (23.0–24.99)	22.4 (0.7)	22.5 (0.4)	
Obese (≥25)	36.5 (0.9)	37.0 (0.6)	
Education (% (SE))			<0.001
Less than middle school	15.7 (0.7)	19.7 (0.6)	
High school graduate	35.3 (0.9)	34.4 (0.6)	
College and more	49.0 (1.1)	45.9 (0.8)	
Family income (% (SE))			0.003
Low	12.9 (0.7)	15.2 (0.5)	
Middle-low	21.8 (0.8)	22.8 (0.6)	
Middle-high	31.6 (0.9)	29.4 (0.8)	
High	33.7 (1.1)	32.6 (1.0)	
Alcohol consumption (% (SE))			<0.001
Never	7.9 (0.4)	7.8 (0.3)	
Past	17.4 (0.7)	16.4 (0.4)	
Current (≤1 time/month)	33.2 (0.9)	30.8 (0.5)	
Current (2–4 time/month)	23.5 (0.8)	23.3 (0.5)	
Current (≥2 time/week)	18.0 (0.7)	21.7 (0.5)	
Smoking status (% (SE))			0.130
Never	58.9 (0.9)	57.8 (0.6)	
Former	25.3 (0.7)	25.0 (0.5)	
Current	15.7 (0.7)	17.2 (0.5)	
Walking rate (% (SE))			0.930
No (<5 day/week, <30 min)	56.0 (1.0)	55.9 (0.7)	
Yes (≥5 day/week, ≥30 min)	44.0 (1.0)	44.1 (0.7)	
Strength training rate (% (SE))			0.194
No (<2 times/week)	74.1 (0.8)	72.9 (0.6)	
Yes (≥2 times/week)	25.9 (1.0)	27.1 (0.6)	
Metabolic syndrome (% (SE))			0.259
No	75.0 (0.7)	74.0 (0.5)	
Yes	25.0 (0.7)	26.0 (0.5)	
Waist (cm)	83.9 ± 0.2	83.9 ± 0.1	0.808
Triglyceride (mg/dL)	128.8 ± 1.7	128.1 ± 1.2	0.730
Elevated triglycerides, %	26.0 (0.8)	24.7 (0.5)	0.167
HDL-cholesterol (mg/dL)			
Male	50.9 ± 0.3	52.1 ± 0.2	0.002
Female	63.1 ± 0.4	63.2 ± 0.3	0.686
SBP (mmHg)	118.3 ± 0.3	118.7 ± 0.2	0.230
DBP (mmHg)	74.5 ± 0.2	74.5 ± 0.1	0.746
Glucose (mg/dL)	99.5 ± 0.4	100.0 ± 0.3	0.210
Elevated fasting glucose, %	33.4 (0.8)	35.3 (0.6)	0.043

Values are presented as survey-weighted means ± SEs for continuous variables and survey-weighted percentages ± SEs for categorical variables. Survey t-tests were used for continuous variables, and Rao–Scott chi-square tests were used for categorical variables.

**Table 2:** Associations between participants’ general characteristics and metabolic syndrome.

Characteristics	No. with MS/no. of Total Adults	ORs ^ǂ^
Sex (% (SE))		
Male	2190/6413	Ref
Female	1864/8355	0.59 (0.51–0.67)
Age (years, mean ± SE)	4054/14,768	1.05 (1.04–1.05)
BMI (kg/m^2^, % (SE))		
Underweight and normal (<23.0)	438/6104	Ref
Overweight (23.0–24.99)	804/3339	3.87 (3.35–4.47)
Obese (≥25)	2812/5325	19.07 (16.81–21.63)
Education (% (SE))		
Less than middle school	1580/3790	Ref
High school graduate	1268/4924	1.09 (0.94–1.26)
College and more	1206/6054	0.93 (0.82–1.05)
Family income (% (SE))		
Low	993/2697	Ref
Middle-low	1064/3562	0.99 (0.84–1.16)
Middle-high	1015/4114	0.94 (0.79–1.11)
High	982/4395	0.89 (0.75–1.06)
Alcohol consumption (% (SE))		
Never	524/1521	Ref
Past	811/2746	0.99 (0.82–1.20)
Current (≤1 time/month)	1067/4557	0.92 (0.77–1.10)
Current (2–4 time/month)	728/3138	0.82 (0.68–1.01)
Current (≥2 time/week)	927/2806	1.12 (0.92–1.37)
Smoking status (% (SE))		
Never	2119/9105	Ref
Former	1161/3497	1.05 (0.90–1.23)
Current	774/2166	1.62 (1.37–1.92)
Walking rate (% (SE))		
No (<5 day/week, <30 min)	2443/8219	Ref
Yes (≥5 day/week, ≥30 min)	1611/6549	0.83 (0.76–0.92)
Strength training rate (% (SE))		
No (<2 times/week)	3246/11,049	Ref
Yes (≥2 times/week)	808/3719	0.58 (0.51–0.65)

ORs: odds ratios, CIs: confidence intervals. **^ǂ^** Adjusted for age, sex, education, family income, BMI, alcohol consumption, smoking status, total energy intake, walking rate and strength training rate.

**Table 3:** The association of revised macronutrient distribution with metabolic syndrome.

Macronutrients	No. with MS/No. of Total Adults	Model 1 ORs	Model 2 ORs
Macronutrients			
Adequate ratio	1101/4176	Ref	Ref
Inadequate ratio	2953/10,592	1.02 (0.91–1.14)	1.01 (0.91–1.13)
Carbohydrate intake ratio			
50–65	1546/6140	Ref	Ref
<50	761/3475	0.92 (0.80–1.06)	0.91 (0.78–1.05)
>65	1747/5153	1.18 (1.05–1.32)	1.14 (1.01–1.30)
Fat intake ratio			
15–30	2259/8273	Ref	Ref
<15	1182/3140	1.26 (1.11–1.43)	1.25 (1.10–1.43)
>30	613/3355	0.86 (0.74–0.99)	0.83 (0.71–0.92)
Protein intake ratio			
10–20	3373/12,060	Ref	Ref
<10	309/997	1.13 (0.92–1.38)	1.07 (0.87–1.32)
>20	372/1711	0.78 (0.66–0.92)	0.78 (0.67–0.92)

ORs: odds ratios, CIs: confidence intervals. Model 1 adjusted for age, sex, education, family income, BMI, alcohol consumption, smoking status, total energy intake, walking rate, and strength training rate. Model 2 included Model 1 covariates and was further adjusted for individual macronutrient intake and fiber intake. The macronutrient corresponding to the exposure was excluded from the covariates to avoid redundant adjustment.

**Table 4:** The association of revised macronutrient distribution with metabolic syndrome by sex.

Macronutrients	Male ORs	Female ORs
Macronutrients		
Adequate ratio	Ref	Ref
Inadequate ratio	1.01 (0.87–1.17)	1.04 (0.89–1.22)
Carbohydrate intake ratio		
50–65	Ref	Ref
<50	0.92 (0.76–1.11)	0.90 (0.69–1.17)
>65	1.08 (0.90–1.29)	1.22 (0.99–1.50)
Fat intake ratio		
15–30	Ref	Ref
<15	1.19 (0.99–1.44)	1.40 (1.16–1.68)
>30	0.90 (0.74–1.11)	0.73 (0.59–0.92)
Protein intake ratio		
10–20	Ref	Ref
<10	1.19 (0.86–1.63)	0.99 (0.76–1.29)
>20	0.80 (0.64–0.99)	0.73 (0.55–0.96)

ORs: odds ratios, CIs: confidence intervals. Adjusted for age, education, family income, BMI, alcohol consumption, smoking status, total energy intake, walking rate, strength training rate, individual macronutrient intake and fiber intake. The macronutrient corresponding to the exposure was excluded from the covariates to avoid redundant adjustment.

**Table 5:** The association of revised macronutrient distribution with metabolic syndrome by age group.

	19–39	40–64	65 Over
	ORs	ORs	ORs
Macronutrients			
Adequate ratio	Ref	Ref	Ref
Inadequate ratio	1.26 (0.93–1.70)	0.94 (0.81–1.09)	1.06 (0.89–1.27)
Carbohydrate intake ratio			
50–65	Ref	Ref	Ref
<50	0.96 (0.68–1.26)	0.87 (0.71–1.06)	1.02 (0.73–1.41)
>65	1.63 (1.08–2.43)	1.09 (0.90–1.31)	0.95 (0.77–1.18)
Fat intake ratio			
15–30	Ref	Ref	Ref
<15	1.62 (1.02–2.59)	1.30 (1.07–1.59)	1.21 (1.00–1.45)
>30	1.32 (0.96–1.80)	0.69 (0.59–0.85)	0.80 (0.58–1.09)
Protein intake ratio			
10–20	Ref	Ref	Ref
<10	1.61 (0.90–2.86)	0.89 (0.66–1.20)	1.15 (0.87–1.53)
>20	0.90 (0.63–1.27)	0.81 (0.64–1.03)	0.67 (0.49–0.92)

ORs: odds ratios, CIs: confidence intervals. Adjusted for age, sex, education, family income, BMI, alcohol consumption, smoking status, total energy intake, walking rate, strength training rate, individual macronutrient intake and fiber intake. The macronutrient corresponding to the exposure was excluded from the covariates to avoid redundant adjustment.

**Table 6:** Isocaloric substitution analysis for metabolic syndrome by sex and age group.

Replacement of Carbohydrate	Total (*n* = 14,768)	Male (*n* = 6413)	Female (*n* = 8355)	19–39 (*n* = 3470)	40–64 (*n* = 6896)	65 over (*n* = 4402)
	ORs	ORs	ORs	ORs	ORs	ORs
With fat, per 5%E	0.94 (0.91–0.97)	0.96 (0.92–0.99)	0.93 (0.88–0.97)	1.01 (0.94–1.10)	0.90 (0.87–0.94)	0.95 (0.91–1.01)
With protein, per 5%E	0.92 (0.86–0.98)	0.88 (0.80–0.96)	0.96 (0.88–1.06)	0.82 (0.71–0.94)	1.02 (0.92–1.12)	0.88 (0.79–0.99)

ORs: odds ratios, CIs: confidence intervals. Adjusted for age, sex, education, family income, BMI, alcohol consumption, smoking status, total energy intake, walking rate, strength training rate, individual macronutrient intake and fiber intake. Sex was excluded from the covariates in sex-stratified analyses, whereas age was retained as a continuous covariate in age-stratified analyses to account for within-group age differences. *n* represents the unweighted number of participants included in each analysis.

## Data Availability

The data presented in this study are available from the KNHANES database at https://knhanes.kdca.go.kr (accessed on 1 July 2026).

## Supplementary Materials

The following supporting information can be downloaded at: https://www.mdpi.com/article/10.3390/nu18162706/s1, Table S1: Specification of multivariable logistic regression models for the associations between macronutrient AMDR categories and metabolic syndrome; Table S2: Sensitivity analysis of the associations between macronutrient intake according to AMDRs and prevalent metabolic syndrome with and without adjustment for BMI; Table S3: Distribution of macronutrient energy percentages in the study population; Table S4: Formal interaction tests for sex and age group in the associations of macronutrient intake with prevalent metabolic syndrome; Table S5: Sensitivity analysis using Benjamini–Hochberg FDR correction for multiple subgroup comparisons; Table S6: Distribution of Participants Across Macronutrient AMDR Categories; Table S7: Weighted prevalence of mutually exclusive macronutrient intake patterns according to the 2025 Korean AMDRs; Table S8: Distribution and association of the number of deviations from the 2025 Korean AMDRs with prevalent metabolic syndrome; Table S9: Distribution of mutually exclusive macronutrient intake patterns and their associations with prevalent metabolic syndrome.

## Abbreviations

The following abbreviations are used in this manuscript:

AMDR	acceptable macronutrient distribution range
BMI	body mass index
CI	confidence interval
FFQ	food frequency questionnaire
HDL	high-density lipoprotein
KDCA	Korea Disease Control and Prevention Agency
KDRIs	Dietary Reference Intakes for Koreans
KNHANES	Korea National Health and Nutrition Examination Survey
MS	metabolic syndrome
NCEP ATP III	National Cholesterol Education Program Adult Treatment Panel III
OR	odds ratio
SE	standard error
