# Supplementary material for: Association Between Revised Macronutrient Intake Ratios and Metabolic Syndrome
Source: Nutrients. 2026 Aug 19;18(16):2706. doi: 10.3390/nu18162706 (PMC13516972; doi:10.3390/nu18162706)
Supplement: Supplementary file 1 [file nutrients-18-02706-s001.zip › nutrients-4467195-supplementary.pdf]

Table S1. Specification of multivariable logistic regression models for the associations between macronutrient AMDR categories and metabolic syndrome.

Exposure	Model	Covariates
Carbohydrate AMDR	Model 1	Age, sex, household income, education, smoking, BMI, alcohol consumption, walking, strength training
Carbohydrate AMDR	Model 2	Model 1 + total energy intake (kcal/day), protein intake (g/day), fat intake (g/day), dietary fiber intake (g/day)
Fat AMDR	Model 1	Age, sex, household income, education, smoking, BMI, alcohol consumption, walking, strength training
Fat AMDR	Model 2	Model 1 + total energy intake (kcal/day), carbohydrate intake (g/day), protein intake (g/day), dietary fiber intake (g/day)
Protein AMDR	Model 1	Age, sex, household income, education, smoking, BMI, alcohol consumption, walking, strength training
Protein AMDR	Model 2	Model 1 + total energy intake (kcal/day), carbohydrate intake (g/day), fat intake (g/day), dietary fiber intake (g/day)

Abbreviations: AMDR, acceptable macronutrient distribution range; BMI, body mass index. Macronutrient AMDR categories were defined as percentages of total energy intake, whereas macronutrient covariates were expressed as absolute daily intakes (g/day). The macronutrient corresponding to the exposure was excluded from the covariates to avoid redundant adjustment.

Table S2. Sensitivity analysis of the associations between macronutrient intake according to AMDRs and prevalent metabolic syndrome with and without adjustment for BMI

Macronutrient	Intake category (%E)	BMI-adjusted model, OR (95% CI)	BMI-excluded model, OR (95% CI)
**Overall macronutrient adequacy**	Adequate	Ref.	Ref.
	Inadequate	1.01 (0.91–1.13)	1.01 (0.92–1.11)
**Carbohydrate**	50–65	Ref.	Ref.
	<50	0.91 (0.78–1.05)	0.91 (0.80–1.03)
	>65	**1.14 (1.01–1.30)**	**1.15 (1.03–1.28)**
**Fat**	15–30	Ref.	Ref.
	<15	**1.25 (1.10–1.43)**	**1.19 (1.05–1.34)**
	>30	**0.83 (0.71–0.92)**	**0.84 (0.74–0.96)**
**Protein**	10–20	Ref.	Ref.
	<10	1.07 (0.87–1.32)	0.98 (0.82–1.18)
	>20	**0.78 (0.67–0.92)**	0.87 (0.76–1.01)

Abbreviations: AMDR, acceptable macronutrient distribution range; BMI, body mass index; CI, confidence interval; OR, odds ratio; %E, percentage of total energy intake.

Values are presented as ORs with 95% CIs estimated using survey-weighted multivariable logistic regression models accounting for the complex sampling design. AMDRs were defined as 50–65%E for carbohydrate, 15–30%E for fat, and 10–20%E for protein. Overall macronutrient adequacy was defined as meeting all three AMDRs, whereas inadequacy was defined as having at least one macronutrient intake outside its corresponding AMDR.

BMI-adjusted model: Adjusted for age, sex, household income, education, smoking status, BMI, alcohol consumption, total energy intake, dietary fiber intake, walking, and muscle-strengthening activity. For each macronutrient-specific analysis, intakes of the other two macronutrients were additionally adjusted for.

BMI-excluded model: Adjusted for the same covariates as the BMI-adjusted model, except for BMI.

Reference categories were 50–65%E for carbohydrate, 15–30%E for fat, and 10–20%E for protein. Bold values indicate statistically significant associations based on 95% CIs excluding 1.00.

Table S3. Distribution of macronutrient energy percentages in the study population

Macronutrient	Mean ± SE	Median	Q1–Q3
Carbohydrate (%E)	57.46±0.16	58.37	49.22-67.07
Protein (%E)	15.46±0.05	14.95	12.65-17.72
Fat (%E)	24.05±0.11	23.19	17.00-30.09

Abbreviations: %E, percentage of total energy intake; SE, standard error; Q1, first quartile; Q3, third quartile.

Values are presented as weighted mean ± SE, weighted median, and weighted interquartile range (Q1–Q3), accounting for the complex survey design of the Korea National Health and Nutrition Examination Survey (KNHANES).

Table S4. Formal interaction tests for sex and age group in the associations of macronutrient intake with prevalent metabolic syndrome.

Analysis	Effect modifier	Macronutrient / theoretical substitution	Wald χ^2	df	P for interaction
Categorical intake	Sex	Carbohydrate intake (<50, 50–65, >65%E)	40.6124	2	**<0.001**
Categorical intake	Sex	Fat intake (<15, 15–30, >30%E)	7.0932	2	**0.029**
Categorical intake	Sex	Protein intake (<10, 10–20, >20%E)	0.6232	2	0.732
Categorical intake	Age group	Carbohydrate intake (<50, 50–65, >65%E)	1.4709	4	0.832
Categorical intake	Age group	Fat intake (<15, 15–30, >30%E)	8.0605	4	0.089
Categorical intake	Age group	Protein intake (<10, 10–20, >20%E)	2.5772	4	0.631
Isocaloric substitution	Sex	Carbohydrate → fat, per 5%E	5.2792	1	**0.022**
Isocaloric substitution	Sex	Carbohydrate → protein, per 5%E	1.7145	1	0.190
Isocaloric substitution	Age group	Carbohydrate → fat, per 5%E	9.9172	2	**0.007**
Isocaloric substitution	Age group	Carbohydrate → protein, per 5%E	7.3498	2	**0.025**

Abbreviation: %E, percentage of total energy intake.

P values for interaction were obtained from joint Wald tests in survey-weighted multivariable logistic regression models. Categorical interaction models included interaction terms between sex or age group and the corresponding macronutrient intake category. In the isocaloric substitution models, interaction terms between sex or age group and continuous fat or protein intake, expressed per 5%E, were evaluated. Bold values indicate *P* for interaction <0.05.

Table S5. Sensitivity analysis using Benjamini–Hochberg FDR correction for multiple subgroup comparisons.

Panel A. Sex- and age-stratified categorical macronutrient analyses

Subgroup	Macronutrient	Comparison	Raw P	FDR-adjusted P
Male	Carbohydrate	<50%E vs. 50–65%E	0.4828	0.6035
Male	Carbohydrate	>65%E vs. 50–65%E	0.9671	0.9818
Female	Carbohydrate	<50%E vs. 50–65%E	0.2245	0.3545
Female	Carbohydrate	>65%E vs. 50–65%E	0.0037	**0.0370**
Male	Fat	<15%E vs. 15–30%E	0.0714	0.1785
Male	Fat	>30%E vs. 15–30%E	0.3229	0.4403
Female	Fat	<15%E vs. 15–30%E	0.0004	**0.0090**
Female	Fat	>30%E vs. 15–30%E	0.0073	0.0548
Male	Protein	<10%E vs. 10–20%E	0.2995	0.4403
Male	Protein	>20%E vs. 10–20%E	0.0435	0.1399
Female	Protein	<10%E vs. 10–20%E	0.9208	0.9818
Female	Protein	>20%E vs. 10–20%E	0.0268	0.1149
19–39 years	Carbohydrate	<50%E vs. 50–65%E	0.9660	0.9818
19–39 years	Carbohydrate	>65%E vs. 50–65%E	0.0513	0.1399
40–64 years	Carbohydrate	<50%E vs. 50–65%E	0.0815	0.1798
40–64 years	Carbohydrate	>65%E vs. 50–65%E	0.2053	0.3422
≥65 years	Carbohydrate	<50%E vs. 50–65%E	0.9818	0.9818
≥65 years	Carbohydrate	>65%E vs. 50–65%E	0.8605	0.9818
19–39 years	Fat	<15%E vs. 15–30%E	0.0428	0.1399
19–39 years	Fat	>30%E vs. 15–30%E	0.0854	0.1798
40–64 years	Fat	<15%E vs. 15–30%E	0.0100	0.0600
40–64 years	Fat	>30%E vs. 15–30%E	0.0006	**0.0090**
≥65 years	Fat	<15%E vs. 15–30%E	0.0504	0.1399
≥65 years	Fat	>30%E vs. 15–30%E	0.1591	0.2808

Subgroup	Macronutrient	Comparison	Raw P	FDR-adjusted P
19–39 years	Protein	<10%E vs. 10–20%E	0.1089	0.2042
19–39 years	Protein	>20%E vs. 10–20%E	0.5366	0.6439
40–64 years	Protein	<10%E vs. 10–20%E	0.4319	0.5633
40–64 years	Protein	>20%E vs. 10–20%E	0.0899	0.1798
≥65 years	Protein	<10%E vs. 10–20%E	0.3218	0.4403
≥65 years	Protein	>20%E vs. 10–20%E	0.0135	0.0675

Bold values indicate FDR-adjusted P <0.05.

Panel B. Sex- and age-stratified isocaloric substitution analyses

Subgroup	Theoretical substitution	Raw P	FDR-adjusted P
Male	Carbohydrate → Fat, per 5%E	0.0370	0.0617
Male	Carbohydrate → Protein, per 5%E	0.0034	**0.0113**
Female	Carbohydrate → Fat, per 5%E	0.0025	**0.0113**
Female	Carbohydrate → Protein, per 5%E	0.4610	0.5763
19–39 years	Carbohydrate → Fat, per 5%E	0.7221	0.7414
19–39 years	Carbohydrate → Protein, per 5%E	0.0050	**0.0125**
40–64 years	Carbohydrate → Fat, per 5%E	<0.0001	**≤0.001**
40–64 years	Carbohydrate → Protein, per 5%E	0.7414	0.7414
≥65 years	Carbohydrate → Fat, per 5%E	0.0758	0.1083
≥65 years	Carbohydrate → Protein, per 5%E	0.0257	0.0514

Bold values indicate FDR-adjusted P <0.05.

P values were obtained from survey-weighted multivariable logistic regression models. To account for multiple subgroup comparisons, the Benjamini–Hochberg false discovery rate (FDR) procedure was applied separately to two families of related hypotheses: 30 sex- and age-stratified comparisons of categorical macronutrient intake (Panel A) and 10 sex- and age-stratified estimates from the isocaloric substitution models (Panel B). The substitution estimates represent the theoretical replacement of 5 percentage points of energy from carbohydrate with the corresponding amount of energy from fat or protein while holding total energy intake and the other macronutrient constant.

Table S6. Distribution of Participants Across Macronutrient AMDR Categories

Macronutrient	AMDR category	n	Weighted %	SE
Carbohydrate	<50%E	3,475	26.69	0.49
	**50–65%E (within AMDR)**	6,140	42.65	0.51
	>65%E	5,153	30.66	0.52
Fat	<15%E	3,140	18.02	0.42
	**15–30%E (within AMDR)**	8,273	56.66	0.48
	>30%E	3,355	25.33	0.49
Protein	<10%E	997	6.43	0.24
	**10–20%E (within AMDR)**	12,060	81.16	0.37
	>20%E	1,711	12.41	0.34

Abbreviations: AMDR, acceptable macronutrient distribution range; %E, percentage of total energy intake; SE, standard error.
Values are presented as unweighted n and weighted percentages with standard errors, accounting for the complex sampling design of the Korea National Health and Nutrition Examination Survey (KNHANES). The AMDRs were defined according to the 2025 Korean Dietary Reference Intakes as 50–65%E for carbohydrate, 15–30%E for fat, and 10–20%E for protein.

Table S7. Weighted prevalence of mutually exclusive macronutrient intake patterns according to the 2025 Korean AMDRs

Carbohydrate (%E)	Fat (%E)	Protein (%E)	n	Weighted %	SE
50–65	15–30	10–20	4,176	**28.7610**	0.4609
50–65	15–30	<10	69	0.5009	0.0622
50–65	15–30	>20	640	4.4496	0.2008
50–65	<15	10–20	114	0.6810	0.0733
50–65	<15	<10	22	0.1443	0.0402
50–65	<15	>20	76	0.5346	0.0764
50–65	>30	10–20	973	7.0265	0.2429
50–65	>30	<10	66	0.5266	0.0709
50–65	>30	>20	4	0.0213	0.0107
<50	15–30	10–20	544	4.1995	0.2099
<50	15–30	<10	66	0.5581	0.0791
<50	15–30	>20	334	2.5002	0.1535
<50	<15	10–20	125	1.0272	0.1002
<50	<15	<10	81	0.5693	0.0705
<50	<15	>20	15	0.0958	0.0286
<50	>30	10–20	1,695	**13.0223**	0.3483
<50	>30	<10	43	0.3248	0.0579
<50	>30	>20	572	4.3926	0.2062
>65	15–30	10–20	2,235	**14.2409**	0.3140
>65	15–30	<10	207	1.4364	0.1137
>65	15–30	>20	2	0.0125	0.0094
>65	<15	10–20	2,198	**12.1989**	0.3412
>65	<15	<10	441	2.3589	0.1382
>65	<15	>20	68	0.4052	0.0550

Carbohydrate (%E)	Fat (%E)	Protein (%E)	n	Weighted %	SE
>65	>30	<10	2	0.0116	0.0092
**Total**			**14,768**	**100.0000**	

Note: %E, percentage of total energy intake; AMDR, acceptable macronutrient distribution range; SE, standard error. The 2025 Korean AMDRs were 50–65%E for carbohydrate, 15–30%E for fat, and 10–20%E for protein. Percentages are weighted estimates accounting for the complex sampling design of the Korea National Health and Nutrition Examination Survey.

Table S8. Distribution and association of the number of deviations from the 2025 Korean AMDRs with prevalent metabolic syndrome

No. of deviations	No. of criteria met	n	Weighted % (SE)	OR (95% CI)
0	3	4,176	28.76 (0.47)	1.00 (Reference)
1	2	4,575	31.10 (0.40)	1.008 (0.889–1.143)
2	1	4,795	31.98 (0.43)	1.037 (0.907–1.185)
3	0	1,222	8.16 (0.25)	0.951 (0.779–1.161)
Total	—	14,768	100.00	—

AMDRs were defined as 50–65%E for carbohydrate, 15–30%E for fat, and 10–20%E for protein. An AMDR deviation was defined as intake below or above the corresponding AMDR. Odds ratios and 95% confidence intervals were estimated using survey-weighted multivariable logistic regression models with participants meeting all three AMDR criteria as the reference group.

Table S9. Distribution of mutually exclusive macronutrient intake patterns and their associations with prevalent metabolic syndrome

Macronutrient pattern	Definition	n	Weighted %	OR (95% CI)
**All within AMDR**	CHO 50–65%E, fat 15–30%E, protein 10–20%E	4,176	28.76	1.000 (Reference)
**High carbohydrate–low fat**	CHO >65%E and fat <15%E, irrespective of protein category	2,707	14.96	**1.295 (1.122–1.495)**
**Low carbohydrate–high fat**	CHO <50%E and fat >30%E, irrespective of protein category	2,310	17.74	**0.809 (0.671–0.976)**
**Isolated high carbohydrate**	CHO >65%E, with fat and protein within AMDR	2,235	14.24	1.052 (0.894–1.237)
**Isolated low carbohydrate**	CHO <50%E, with fat and protein within AMDR	544	4.20	1.054 (0.817–1.361)
**Isolated high fat**	Fat >30%E, with CHO and protein within AMDR	973	7.03	0.905 (0.723–1.132)
**Isolated protein deviation**	Protein <10%E or >20%E, with CHO and fat within AMDR	709	4.95	0.919 (0.730–1.158)
**Other mixed/deviation patterns**	Other combinations not classified above	1,114	8.12	0.943 (0.772–1.152)
**Total**		**14,768**	**100.00**	

Global P for macronutrient pattern = 0.0003

Values are presented as unweighted numbers and survey-weighted percentages. Odds ratios (ORs) and 95% confidence intervals (CIs) were estimated using survey-weighted multivariable logistic regression models. The reference group comprised participants whose carbohydrate, fat, and protein intakes were all within the acceptable macronutrient distribution ranges (AMDRs). Models were adjusted for age, sex, household income, education, smoking status, body mass index, alcohol consumption, total energy intake, dietary fiber intake, walking, and muscle-strengthening activity. The overall association of the mutually exclusive macronutrient patterns with prevalent metabolic syndrome was evaluated using a Type 3 Wald test (Wald χ^2 = 27.13, df = 7, P = 0.0003). AMDRs were defined as 50–65% of energy (%E) from carbohydrate, 15–30%E from fat, and 10–20%E from protein. Bold values indicate 95% CIs that do not include 1.00.
